# Validation of selection signatures for coat color in the Podolica Italiana gray cattle breed

**DOI:** 10.3389/fgene.2024.1453295

**Published:** 2024-12-09

**Authors:** Silvia Bruno, Giacomo Rovelli, Vincenzo Landi, Fiorella Sbarra, Andrea Quaglia, Fabio Pilla, Emiliano Lasagna, Elena Ciani

**Affiliations:** ^1^ Dipartimento di Bioscienze, Biotecnologie e Ambiente, Università degli Studi di Bari “Aldo Moro”, Bari, Italy; ^2^ Dipartimento di Scienze Agrarie, Alimentari e Ambientali, Università degli Studi di Perugia, Perugia, Italy; ^3^ Dipartimento di Medicina Veterinaria, Università degli Studi di Bari “Aldo Moro”, Bari, Italy; ^4^ Associazione Nazionale Allevatori Bovini Italiani da Carne (ANABIC), Perugia, Italy; ^5^ Dipartimento di Agricoltura, Ambiente e Alimenti, Università degli Studi del Molise, Campobasso, Italy

**Keywords:** pigmentation, coat color, cattle, hair graying, selection signatures, single-nucleotide polymorphisms

## Abstract

Taurine and indicine gray cattle represent relevant livestock resources in many countries of the world. A gray coat color and pigmented skin, which are common in most of the gray cattle breeds, have been demonstrated to confer better adaptation to solar radiation and thermal stresses. In a previous study adopting the F_ST_-outlier approach with BayeScan v2.0, we identified differentially selected genomic regions in a set of gray cattle breeds, including the Podolica Italiana, and contrasted these findings with four non-gray cattle breeds. More supported signals were detected on bovine chromosomes (BTAs) 2, 4, 14, and 26 that encompassed more than fifty genes known to be directly or indirectly related to one or more steps in pigment biology. In the present study, we aimed to validate the previously observed signals using the same methodological approach on three new Podolica Italiana sample sets (N = 30 animals each). These animals were selected from the ANABIC genetic station during performance tests as being representative of the Podolica Italiana population at three different timeframes separated by approximately 10 years each. We typed these samples to the loci of 23,027 quality-controlled single-nucleotide polymorphisms. We also analyzed the dataset using the haplotype-based approach available in hapFLK v1.4 software. Both the F_ST_-outlier and hapFLK approaches validated the abovementioned signals on BTAs 2, 4, 14, and 26. Moreover, both methods detected additional supported regions on BTAs 7 and 18 that included a total of 42 genes, of which most were already known from literature to be implicated in pigmentation traits.

## Introduction

In wild animal species, coloration is considered as one of the mechanisms of camouflage, intra- and inter-specific communications, sexual selection, as well as a range of physiological and physical functions, including reflecting or absorbing radiation and/or providing a surface to enhance or reduce evaporation (Caro, 2005; Caro and Mallarino, 2020). Consequently, adaptive evolutionary pressures on wild animal coloration are generally accepted despite their unclear mechanisms because scientific evidence of these criteria require difficult experimental approaches (Caro, 2005). Although model species have been the main resources for studying the genetic basis of animal coloration for decades, recent genome-wide genotyping technologies have opened new avenues for such studies in more number of species with different color traits (San-Jose and Roulin, 2017). Most color traits can be considered quantitative as they exhibit continuous phenotypic variations, and the expressions of such traits are affected by various environmental factors in addition to being strongly determined by genetic factors.

In domestic animal species, empirical human-driven selection of coat color has been shown to have occurred since early times after domestication (Fang et al., 2009; Ludwig et al., 2009; Cieslak et al., 2011; Linderholm and Larson, 2013; Ollivier et al., 2013). By carefully selecting rare genetic mutations and potentially even purifying natural selection in the wild counterparts, humans have frequently ensured the retention of coat colors and patterns in domestic animals that were unusual in the wild progenitors. Indeed, living under human care has exposed domestic animals to lower selective constraints on coat color, and even phenotypes that are strongly linked to pathogenic effects can be positively selected by breeders (Cieslak et al., 2011). Several reasons may have prompted early animal breeders to select coat color variations in domestic animals over wild ones, including esthetic preferences for novelty, selection for reduced camouflage to facilitate animal husbandry, and/or distinguish the domesticated forms from their wild ancestors (Fang et al., 2009). Coloration has often played a relevant role in breed formation, especially in cattle, with a single type of coat color and/or pattern being fixed in many breeds and some breeds even being named on the basis of their coat color.

One of the coat colors typical to many taurine and indicine cattle breeds is pale gray, which is acquired by young growing animals that were fawn colored at birth. The typical diluted gray coat observed in these animals and eumelanic pigmented skin are considered to confer better thermoregulation abilities compared to darker coats owing to higher light reflectance, while dark skin would still offer protection against ultraviolet radiation (da Silva et al., 2003). In our recent work, we used medium-density single-nucleotide polymorphism (SNP) array genotype data and applied a multicohort F_ST_-outlier approach to compare fifteen taurine gray cattle breeds with four non-gray cattle breeds (Angus, Limousin, Charolais, and Holstein); we detected more than 50 candidate genes for selection, all of which were almost directly or indirectly involved in pigmentation, while some were already known for their roles in phenotypes related to hair graying in mammals (Senczuk et al., 2020). A similar multicohort F_ST_-outlier approach was adopted for pairwise contrast between seven gray indicine cattle breeds and both taurine and indicine non-gray cattle breeds (Senczuk et al., 2022), where most of the signals were common with those obtained in the abovementioned study on taurine cattle.

In the present study, our goal was to validate the status of selection signatures for the discovered candidate variants using medium-density SNP array genotype data and the multicohort F_ST_-outlier approach to compare an independent set of gray cattle belonging to an Italian breed with four non-gray taurine cattle breeds (Angus, Limousin, Charolais, and Holstein) considered in previously studies by Senczuk et al. (2020, 2022). The population dataset was arranged in three cohorts representing three time periods ranging from 1995 to 2019 to account for the evolution of the coat color in the considered breed.

## Material and methods

### Breeds and pigmentation phenotypes

Podolica Italiana is a local cattle breed that is mainly reared in continental Southern Italy and often under almost year-long grazing on natural grasslands. As of the end of 2023, the breed had 37,312 animals registered in the herd book of the National Association of Italian Beef Cattle Breeders (ANABIC) (ANABIC, 2024). This breed has an ancient origin and has been generally considered to have descended from the “Podolian” strain that arrived in Italy from Anatolia and the Fertile Crescent during the Etruscan migration in historical times (third to fifth century *anno domini*) (Di Lorenzo et al., 2018; Colombi et al., 2024). Nevertheless, the origin of this breed remains controversial, with numerous studies highlighting an admixed taurine-indicine origin for European cattle belonging to the Podolian trunk, including Podolica Italiana (Upadhyay et al., 2017; Barbato et al., 2020; Mastrangelo et al., 2020; Senczuk et al., 2021).

Podolica Italiana is a very rustic and frugal breed that is well-adapted to harsh geoclimatic conditions, such as the cold and snowy winters in the pasture-wooded areas on the mountainous plateau of Sila (Calabria) as well as the hot and dry summers in the karst pasture areas (“Murge”) of Apulia. It is renowned for producing low-quantity but high-quality milk mainly for the production of “Caciocavallo Podolico”, a mature cheese recognized as a slow food presidium (https://www.fondazioneslowfood.com/it/) and included among the Italian traditional agri-food products (PAT; https://www.gazzettaufficiale.it/eli/gu/2020/02/20/42/so/9/sg/pdf). This breed has also been shown to produce meat that is naturally characterized by a beneficial content of polyunsaturated fatty acids (PUFAs), omega-3 fatty acids, and linoleic conjugated acids (CLAs) (Tarricone et al., 2011; Marino et al., 2014). The phenotypic trait of interest for this study is the distinctive coat color of Podolica Italiana (referred here as “gray”) that is shared by other Podolian-derived cattle breeds and characterized by the presence of a wheat-coated pigmentation of the calf at birth, a light gray coat color at puberty, followed by the development of a eumelanic coat color in some body areas of the adult animals (mainly male subjects).

From an investigation carried out among the Podolica Italiana breeders and ANABIC genetic center staff, it was found that the coat color has been darkening and becoming close to black over the past two decades. Although there is no documented literature for this phenomenon, it appears that a possible positive selection pressure exists on darker animals as a darker color is currently preferred by farmers as a breed distinction attribute compared to other Italian cattle breeds belonging to the Podolian trunk (notably Chianina, Marchigiana, Romagnola, and Maremmana). The four non-gray breeds investigated in this study were selected as representatives of the following coat color phenotypes: solid black (Angus), solid white (Charolais), solid red (Limousin), and black and white piebald (Holstein).

### Animal sampling

A total of 90 blood samples from elite young bulls representing a subset of the Podolica Italiana elite bulls were collected from the genetic station of ANABIC located in Laurenzana, Potenza, during the performance tests from 1995 to 2019 (Lasagna et al., 2015; Rovelli et al., 2020). These 90 selected animals were representative of the “gray” phenotype and were divided in three groups of 30 animals each based on the year of birth as follows: i) Podolica_old (POD_OLD) animals born from 1995 to 1997; ii) Podolica_medium (POD_MED) animals born in 2009; iii) Podolica_new (POD_NEW) with animals born in 2019. The sampled animals were distributed into these three groups (POD_OLD, POD_MED, and POD_NEW) to assess whether similar or somehow dissimilar patterns of selection signatures could be detected as a result of the evolution of the Podolica Italiana genetic makeup that was also possibly driven by recent farmer preferences toward darker phenotypes.

Individual blood samples were collected from the jugular veins of young bulls during the performance tests. The sampling process adhered to the guidelines of the Food and Agriculture Organization (FAO) for characterization of animal genetic resources and animal management in accordance with the criteria defined in the Welfare Quality Project (WQP) 53. The ANABIC Central Technical Committee of the National Herd Book approved all the activities in 2020 by considering all aspects involved in the blood collection, management, and handling of the animals. Blood sampling was performed by trained veterinarians by strictly following the standard procedures and relevant national guidelines to ensure appropriate animal care. The present research conformed with the ARRIVE guidelines and regulations (https://arriveguidelines.org). The samples were collected in EDTA K3-coated vacuum tubes and stored at −20°C before DNA extraction.

### Genomic DNA extraction and high-throughput genotyping

The genomic DNA was purified using the GenElute Blood Genomic DNA kit (Sigma Aldrich, St. Louis, MO, United States) as described in Sarti et al. (2019). All bulls were genotyped using the GeneSeek Genomic Profiler Bovine LDv4 33K chip (Illumina Inc., San Diego, CA, United States) that contains 30,111 SNPs. The samples were processed at the Agrotis laboratory (LGS, Cremona, Italy) using standard multisample protocols and reagents according to manufacturer instructions (Khatkar et al., 2012). This chip was used as it is the official array used by ANABIC to genotype all the young bulls evaluated in the performance tests. The genotypes of the four non-gray breeds (Charolais, Limousin, Holstein, and Angus) used as comparisons to Podolica Italiana were obtained from the dataset reported by Senczuk et al. (2020). The following quality control criteria were applied in this study: (i) loci with call rates ≤90% (i.e., locus missingness >10%), (ii) loci with minor allele frequency ≤1%, and (iii) individuals with genotyping rates ≤90% (i.e., individual missingness >10%) were excluded from the study; (iv) non-autosomal loci were removed.

### Detection of *F*_ST_-outlier markers

Using the Markov chain Monte Carlo (MCMC) model, the BayeScan v2.1 software (Foll and Gaggiotti, 2008) generates F_ST_ null distributions under neutral expectations that allow testing of significant departures from neutrality. In this study, we adopted the F_ST_-outlier approach available in BayeScan v2.1 software to identify the SNP loci under differential selection in the gray and non-gray cattle breeds. Accordingly, we conducted pairwise comparisons of the three Podolica Italiana subgroups (POD_OLD, POD_MED, and POD_NEW) with each of the four non-gray breeds (Table 1). For each pair of gray and non-gray breeds, the loci that showed *p*-values < 0.05 were retained. Then, we applied a false discovery rate (FDR) correction for multiple tests (Benjamini and Hochberg, 1995) to these results using R software v4.0.3 (R Core Team, 2018), where the significance threshold was set at *q*-value = 0.05. The FDR-corrected significant markers were considered as “putatively subjected to differential selection”. In the results, we first provide a descriptive overview of the findings and then specifically check if the loci previously detected as significant by Senczuk et al. (2020) are also significant in our study (validation); finally, we search for possible novel signals of selection. For the latter, we focused on those loci that appeared to be significant in at least 7 out of 12 pairwise contrasts (Supplementary Table S1) to reduce false positive signals.

**TABLE 1:** Results of the comparisons between BayeScan and HapFLK analyses performed in this study and the 12 significant loci detected in at least 15 pairwise contrasts out of 60 by Senczuk et al. (2020).

BTA	SNP ID^a^	rsID	BayeScan results^b^	HapFLK results^c^
2	Hapmap49624-BTA-47893	rs41638273	11	3
4	Hapmap53144-ss46525999	rs41255147	6	3
14	BTB-01532239	rs42649775	12	3
14	BTB-01530788	rs42646660	12	3
14	BTB-00557532	rs41724332	12	3
14	Hapmap46986-BTA-34282	rs41627953	11	3
14	Hapmap46735-BTA-86653	rs41657755	9	3
14	ARS-BFGL-NGS-36089	rs110774011	9	3
14	Hapmap30932-BTC-011225	rs41724536	6	3
*14*	*BTB-01280026*	*rs42404006*	*1*	*1*
*14*	*Hapmap27934-BTC-065223*	*rs42403970*	*1*	*1*
26	ARS-BFGL-NGS-11271	rs110513274	9	3

^a^ List of loci detected as significant in at least 15 pairwise contrasts out of 60 by Senczuk et al. (2020).

^b^ Number of BayeScan pairwise contrasts where each locus was detected as significant (in one or more of the 12 contrasts) in our study.

^c^ Number of HapFLK contrasts where each locus was detected as significant (in one or more of the three contrasts) in our study.

### Detection of selective signals with hapFLK

As a complementary approach to detect selective signatures, we used hapFLK statistics (Fariello et al., 2013); the hapFLK approach is built on FLK statistics (Bonhomme et al., 2010), which is an extension of the original Lewontin and Krakauer (LK) test. The FLK test uses a phylogenetic estimation of the population kinship matrix to account for the historical branching and heterogeneity of genetic drift. Similar to FLK, hapFLK incorporates the hierarchical structure of populations but the test is extended to account for the haplotype structure in the multiple-populations sample. It is noted that this method has been shown to be especially robust against bottleneck and migration effects (Manunza et al., 2016). In our hapFLK analysis, the number of haplotype clusters was set to 20 using the cross-validation procedure assumed in the fastPHASE19 model, and the hapFLK statistics was calculated as the average of 30 iterations of expectation maximization (Scheet and Stephens, 2006). The analyses were conducted for the three sets of animal groups (POD_OLD vs. all non-gray, POD_MED vs. all non-gray, and POD_NEW vs. all non-gray) and raw *p*-values were calculated from the null distribution of the empirical values. The FDR filter was used to correct for multiple tests (Benjamini and Hochberg, 1995), and the significance threshold was set at *q*-value = 0.05. Similarly to the results of the BayeScan analysis, we then provide a descriptive overview of the obtained results and specifically check if the loci previously detected as significant by Senczuk et al. (2020) are also significant in this study; finally, we search for possible novel signals of selection. For the latter, we focused on those loci that appeared to be significant in at least 2 out of 3 contrasts to reduce false positive signals.

### Gene content of regions under selection and network analysis

To query the most updated assembly, the positions of the SNPs in the putatively selected regions were manually updated from the UMD_3.1.1 to ARS-UCD2.0 assembly by blasting the UMD_3.1.1 sequences harboring each significant SNP against the ARS-UCD2.0 reference genome using the online NCBI BlastN option. The regions that were putatively under selection were defined from a range of ±250 kbps upstream and downstream of each FDR-corrected significant locus. The window size was arbitrarily defined using the same experimental conditions that we used previously (Senczuk et al., 2020, 2022) and by considering that some studies have suggested the use of large intervals to account for (i) enhancers and repressors that may be as distant as 500 kbps from their genes (Wang et al., 2007) and (ii) SNPs that may be markers for structural variations affecting large chromosomal segments (Hinds et al., 2006). In the case of overlapping ranges, we considered the overall interval as a single region. The annotated genes within the genomic regions putatively under selection were obtained by querying the NCBI *Bos taurus* Genome Data Viewer using the reference genomes from Bos_Taurus_ARS-UCD2.0 (https://www.ncbi.nlm.nih.gov/gdv/browser/genome/?id=GCF_002263795.3). To investigate the biological functions and phenotypes known to be affected by each annotated gene, we conducted (i) a comprehensive search of the available literature and public databases and (ii) a gene ontology (GO) analysis on the candidate genes preliminarily identified using the F_ST_-outlier and hapFLK approaches as well as those previously identified by Senczuk et al. (2020) with the GOnet online tool (Pomaznoy et al., 2018).

## Results

After implementing the quality control steps, the final dataset included a total of 23,027 SNP loci from the 90 animals.

### BayeScan analysis

**FIGURE 1:**
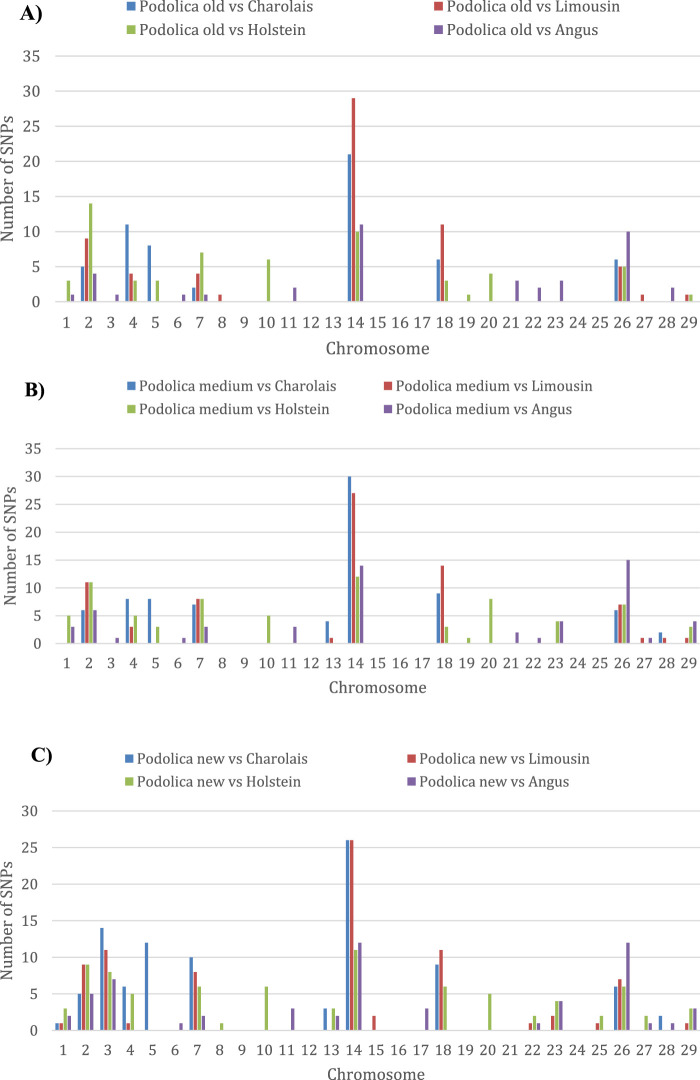
Histograms showing the numbers of significant loci detected per chromosome in the considered pairwise contrasts. Comparisons of the test breeds **(A)** POD_OLD, **(B)** POD_MED, and **(C)** POD_NEW with four non-gray reference breeds.

The overall results of the differential selection signatures study based on the F_ST_-outlier approach implemented in BayeScan with a *q*-value of 0.05 and by contrasting each Podolica Italiana subgroup (POD_OLD, POD_MED, and POD_NEW) with each of the four non-gray breeds are reported in Supplementary Table S2. For each of the twelve pairwise contrasts, the per-chromosome counts of the significant loci (*q*-value < 0.05) are shown in Supplementary Table S3. Overall, increasing proportions of signals are observed for each pairwise contrast when moving from POD_OLD to POD_MED to POD_NEW. The lowest numbers of significant loci were always observed in the pairwise contrasts involving the three Podolica Italiana subgroups against the Angus reference breed. The chromosome harboring the highest number of significant loci was the bovine chromosome (BTA) 14 for all three subgroups (Figure 1; Supplementary Table S3).

### HapFLK analysis

**FIGURE 2:**
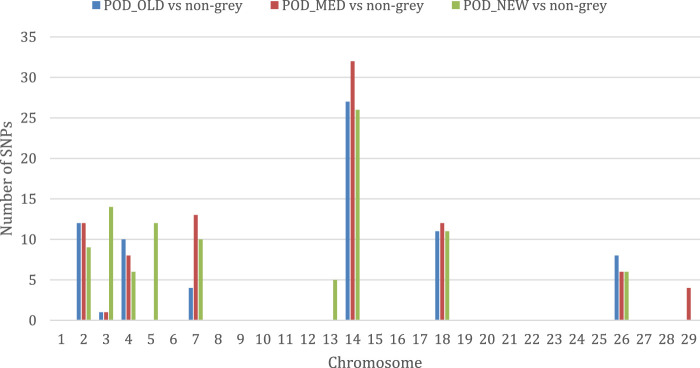
Histogram displaying the number of significant loci detected per chromosome in the comparisons between the three Podolica Italiana subgroup test breeds and the four non-gray reference breeds.

The results of the selection signatures study based on HapFLK statistics obtained by comparing each Podolica Italiana subgroup (POD_OLD, POD_MED, and POD_NEW) with the multiple-populations sample including all four non-gray reference breeds are reported in Supplementary Table S4. Overall, we obtained a total of 260 significant loci (*q*-value < 0.05). In particular, we detected 73 signals in the comparison of POD_OLD with non-gray, 88 signals in the comparison of POD_MED with non-gray, and 99 signals in the comparison of POD_NEW with non-gray (also found from the BayeScan analysis) breeds. Considering the number of per-chromosome significant loci detected in each comparison (Figure 2), the chromosomes harboring the highest numbers of signals, as also observed from the BayeScan analyses (Figure 1), were BTAs 2, 4, 7, 14, 18, and 26, with the maximum number of signals observed for BTA 14.

### Validation of known selection signatures

In a previous study by Senczuk et al. (2020), the outlier loci related to coat color were investigated using the robust multicohort approach based on pairwise comparisons between gray and non-gray taurine cattle breeds. The 12 most supported signals were detected on BTAs 2 (Hapmap49624-BTA-47893), 4 (Hapmap53144-ss46525999), 14 (with nine significant SNP loci), and 26 (ARS-BFGL-NGS-11271). Here, we compared our results obtained from both the BayeScan and HapFLK analyses with those reported by Senczuk et al. (2020) to validate the previous observations. The 12 loci identified as highly supported (i.e., loci detected as significant in at least 15 pairwise comparisons out of 60 performed) by Senczuk et al. (2020) are reported in Table 1, and the results obtained for these loci in our differential selection signatures analysis using both BayeScan and HapFLK software are shown. Out of these, only two signals that are both located on BTA 14 (in italics in Table 1) appear to be poorly supported by our analyses, while all remaining signals are supported by at least half of the total number of comparisons.

### Identification of novel selection signatures

Overall, a total of 828 significant loci were detected from the 12 (3 × 4) pairwise comparisons performed using BayeScan software. Out of these, 69 were unique (i.e., observed in a single pairwise contrast) (Supplementary Table S2) while 22 were observed in at least 7 comparisons out of 12 (Supplementary Table S5) and 7 were arbitrarily selected as having minimum multiple-occurrence (>50%) threshold. Of the 260 significant loci detected through HapFLK analysis, 77 were unique (i.e., observed in a single contrast), 21 were observed in 2 out of 3 contrasts, and 47 were observed in 3 out of 3 contrasts. Similar to the findings from the BayeScan analysis, the minimum multiple-occurrence threshold for HapFLK analysis was set to at least 2 (>50%) (Supplementary Table S6).

Based on joint consideration of the two thresholds, a total of 5 novel loci (2 on BTA 7 and 3 on BTA 18) putatively under selection were identified (Table 2). The presence of genes relevant to the investigated phenotype was checked in the interval ±250 kbps upstream and downstream of each significant locus after manually updating the positions of all significant signals to the ARS-UCD2.0 current reference genome. In particular, two regions were identified from overlapping ranges, one on BTA 7 (45,418,300–45,941,037 bps) and another on BTA 18 (13,819,857–14,607,607 bps). The region on BTA 7 included 9 genes while that on BTA 18 included 36 genes (Table 2). The possible involvements of these genes in pigmentation are described in Supplementary Tables S1, S2.

**TABLE 2:** Results of both BayeScan and HapFLK analyses showing the newly identified highly supported loci and genes located in the corresponding intervals.

BTA	SNP ID	rsID	Position (bp)^a^	N. BayeScan^b^	N. HapFLK^c^	Considered interval (bp)^d^	Genes^e^
7	ARS-BFGL-NGS-12557	rs43513971	45,668,300	9	3	45,418,300 to 45,941,037	*C7H5orf15, LOC132345820, VDAC1, TCF7, SKP1, PPP2CA, LOC112447418, MIR2285DI,* and *CDKL3*
7	ARS-BFGL-NGS-20141	rs109739739	45,691,037	7	3		
18	ARS-BFGL-NGS-4996	rs108991316	14,069,857	7	3	13,819,857 to 14,607,607	*LOC517901, ZC3H18, CYBA, MVD, LOC132342792, SNAI3, RNF166, CTU2, PIEZO1, MIR2327, CDT1, APRT, GALNS, TRAPPC2L, PABPN1L, CBFA2T3, LOC132342793, ACSF3, CDH15, SLC22A31, ANKRD11, LOC132342794, LOC112442271, SPG7, RPL13, LOC112442426, CPNE7, DPEP1, CHMP1A, SPATA33, CDK10, LOC104974758, SPATA2L, VPS9D1, ZNF276,* and *FANCA*
18	ARS-BFGL-NGS-31386	rs110266670	14,164,337	7	3		
18	Hapmap44238-BTA-42338	rs41635461	14,357,607	9	3		

^a^ Position of the SNP in the ARS-UCD2.0 reference genome.

^b^ Number of significant pairwise contrasts in which each locus was detected in the BayeScan analysis.

^c^ Number of significant contrasts in which each locus was observed in the HapFLK analysis.

^d^ Interval used for the gene search in the range of ±250 kbps upstream and downstream of each significant locus.

^e^ Genes located in the identified regions using the ARS-UCD2.0 bovine genome assembly.

### GO analysis

GO analyses were performed using the GOnet web application (Pomaznoy et al., 2018) on the list of 113 genes obtained by merging (i) genes identified previously by Senczuk et al. (2020) and (ii) newly identified genes in the regions on BTAs 7 and 18. Notably, GOnet reconstructed the relationships of the input genes to the GO terms by grouping the genes sharing a common biological process. Overall, we identified a total of 62 biological processes in which the investigated genes were involved (Supplementary Table S7). Of these, some GO terms were related to the principal stages of pigmentation, including (i) differentiation and development of melanocytes from embryonic neural crest cells (n = 12), (ii) pigment synthesis in the melanocytes (n = 6), (iii) melanin transfer from melanocytes to keratinocytes (n = 14), (iv) responses to different classes of external stimuli (n = 2), and (v) autophagy and lysosomal disorders (n = 7). Finally, 21 GO terms were found to be related to other biological processes that are not directly involved in pigmentation.

## Discussion

In the present study, we provide independent evidence for signals of differential selection between gray and non-gray cattle breeds that were previously detected from comparisons of fifteen taurine (Senczuk et al., 2020) and seven indicine (Senczuk et al., 2022) cattle breeds with four cattle breeds representing non-gray coat colors: solid black (Angus), solid white (Charolais), solid red (Limousin), and black and white piebald (Holstein). By applying the same multicohort F_ST_-outlier approach reported in Senczuck et al. (2020, 2022) to a new dataset of medium-density genome-wide SNP genotypes from the Podolica Italiana gray cattle breed, we support and validate 10 (out of 12) candidate SNP loci on BTAs 2 (Hapmap49624-BTA-47893), 4 (Hapmap53144-ss46525999), 14 (BTB-01532239, BTB-01530788, BTB-00557532, Hapmap46986-BTA-34282, Hapmap46735-BTA-86653, ARS-BFGL-NGS-36089, Hapmap30932-BTC-011225), and 26 (ARS-BFGL-NGS-11271). Interestingly, the above results were obtained despite using an SNP array format (GeneSeek Genomic Profiler Bovine LDv4 33K chip) different from the one used previously by Senczuck et al. (2020, 2022), i.e., the Illumina BovineSNP50 Genotyping BeadChip, thus strengthening the reported evidence.

Podolica Italiana has been traditionally classified as a taurine cattle breed. However, diversity studies based on genome-wide SNP data (Upadhyay et al., 2017; Mastrangelo et al., 2020; Barbato et al., 2020; Senczuk et al., 2021) have highlighted a certain degree of post-domestication indicine introgression in Podolica Italiana as well as other gray Italian (e.g., Chianina, Marchigiana, Romagnola, Maremmana) and non-Italian (e.g., Turkish gray and Hungarian gray) cattle breeds (Upadhyay et al., 2017; Mastrangelo et al., 2020). This observation is consistent with the hypothesis of a single origin for the genetic mechanisms underlying the gray coat color phenotype in both cattle subspecies, possibly representing an ancestral condition in the early genetic stock of Asiatic indicine origin while being a posterior acquisition in the taurine cattle breeds (Senczuk et al., 2022). The positive roles of this novel and distinct adaptation-related phenotype on environmental robustness (i.e., higher thermotolerance and lower UV-induced damage due to higher solar reflectance) and esthetic preferences may have been relevant drivers for subsequent and continued positive selection pressures in taurine cattle breeds.

In the present study, we also identified selection signals (on BTAs 7 and 18) that were not previously highlighted by Senczuk et al. (2020, 2022). Consistent with the robust multicohort approach adopted herein, which is expected to maintain a low risk of false positives, the genomic regions putatively under differential selection pressure in BTAs 7 and 18 harbor several candidate genes that are known to be directly or indirectly related to pigmentation phenotypes. Notably, in mammals, the voltage dependent anion channel 1 (*VDAC1*) has a prominent role in maintaining intracellular ${Ca}^{2+}$ homeostasis (Shoshan-Barmatz et al., 2010; Shoshan-Barmatz and Ben-Hail, 2012): a process that has been shown to be crucial for melanogenesis (Zang et al., 2019; Jia et al., 2020; Le et al., 2021; Wang et al., 2022). Transcription factor 7 (*TCF7*) is involved in the formation of a complex with beta-catenin and activation of transcription through the Wnt/beta-catenin signaling pathway, where Wnt signaling is involved in regulating the differentiation of neural crest cells into melanocytes (Patapoutian and Reichardt, 2000); its role in melanocyte expansion and differentiation is well-known in mice (Dunn et al., 2000). S-phase kinase associated protein 1 (*SKP1*) encodes an adaptor component of the SKP1-CUL1-RBX-F-box protein (SCF) E3 ubiquitin-protein ligase complex. Liu et al. (2023) demonstrated that the SKP1–CUL1–F-box protein complex related genes in *Nelumbo* play an important role in the biosynthesis of anthocyanins, a wide class of plant pigments (Grotewold, 2006). Protein phosphatase 2 catalytic subunit alpha (*PPP2CA*) encodes the catalytic subunit of 2A phosphatase, whose knockout in mice has been shown by Fang et al. (2016) to be associated with visible melanin deposition and pigmentation at the base of the claws and in paws compared to wild type mice. Cytochrome B-245 alpha chain (*CYBA*) is associated with *NOX2* to form the redox element cytochrome B expressed mainly in phagocytes and responsible for generating reactive oxygen species (ROS) starting from O_2_. Liu et al. (2012) showed that ROS can modulate melanin expression in mouse melanoma cells. Acyl-CoA synthetase family member 3 (*ACSF3*) has been shown through a recent study contrasting the transcriptional profiles of different coat colors in soybean seeds (yellow vs. black) to be four times more expressed in black seed coats, suggesting its role in the black pigmentation of soybean seeds (Kafer et al., 2023). Cadherin 15 (*CDH15*) has been found in a selection signature contrasting Hainan Black goats with white Alashan Cashmere goats (Chen et al., 2022). Ankyrin repeat domain containing 11 (*ANKRD11*) has been shown to be involved in pigmentation in cattle as some SNPs in this gene were identified to be considerably divergent between black and red Angus cattle (He et al., 2022). Moreover, it has been shown that mice with a homozygous neural-crest-specific deletion of *ANKRD11* have severe craniofacial phenotypes, including loss of black pigment on the nose (Roth et al., 2021). Ribosomal protein L13 (*RPL13*) was found to be expressed in human melanoma melanocytes, with advanced-stage melanoma cell lines having four to six times greater *RPL13* protein expressions (Kardos et al., 2014).

In humans, an intronic deletion of charged multivesicular body protein 1A (*CHMP1A*) was reported by Farré et al. (2023) to be significantly associated with red hair. Spermatogenesis associated 3 (*SPATA33*) has been identified as a putative melanoma susceptibility gene (Fang et al., 2020); it is also associated with Fanconi anemia, a rare genetically heterogeneous recessive disorder with variable clinical manifestations, including cutaneous pigmentary alterations such as widespread areas of hyper- and hypo-pigmentation of the skin in characteristic patterns and café-au-lait spots (Ogilvie et al., 2002; Ruggiero et al., 2021). In addition, *SPATA33* has been associated with facial pigmentation spots (Jacobs et al., 2015) and/or with red hair phototype (Farré et al., 2023). Cyclin dependent kinase 10 (*CDK10*) was shown to be significantly associated with hair color in humans (Lona-Durazo et al., 2021); it has also been suggested as a putative relevant gene associated with vitiligo, a multifactorial polygenic disorder characterized by acquired depigmented skin and overlying hair resulting from the destruction of melanocytes (Cai et al., 2021). Spermatogenesis associated 2 like (*SPATA2L*) was reported by Cai et al. (2021) as a newly putative functional gene associated with vitiligo susceptibility.

A study by Bonilla et al. (2021) on DNA methylation (DNAm) as a potential mediator between pigmentation genes, pigmentary traits, and skin cancer showed that *SPATA2L* had a potential pleiotropic association with the phenotypes of skin color and black hair since these traits colocalized with the DNAm site near the melanocortin 1 receptor (*MC1R*) gene are known to be responsible for coat color variations in cattle (Goud et al., 2021). Zinc finger protein 276 (*ZNF276*) was identified by Zhang et al. (2020) as a selection signature in a Chinese native pig characterized by its black coat color. Moreover, it has been found to be associated with red hair in humans (Farrè et al., 2023). Mutations in the Fanconi anemia complementation group A (*FANCA*) gene are the most common cause of Fanconi anemia (Ogilvie et al., 2002; Ruggiero et al., 2021). Moreover, by contrasting red vs. black Angus cattle, He et al. (2022) showed that there were three consecutive strongly associated SNPs in the *FANCA* gene that considerably diverged between the two cattle breeds.

Premature graying of hair has been observed in many taurine and indicine breeds as a complex phenotype. Indeed, while the calves display wheat-coated pigmentation associated with a paler ventral coloration (pigmentation patterning) at birth, this coat color becomes light gray at puberty, followed by subsequent development to a eumelanic coat color in some body areas at adulthood mostly in male subjects (sexual dimorphism). As such, it is not surprising that many of the candidate genes identified in this study, either from the new or validated selection signals, were found in the GO analysis to play roles at disparate stages of coat color/pattern formation.

Our chronologically tripartite Podolica Italiana dataset allowed us to highlight a temporal evolution in the selection signature patterns. However, the observed patterns did not apparently correlate with known coat color evolution trends experienced by the breed over the past decade. Indeed, this breed is known to have become more eumelanic than observed in the past. Under the not-necessarily true assumption that such coat color darkening of Podolica Italiana may imply mechanisms that make this breed genetically closer to the black Angus cattle in our dataset, one would expect that when moving from the oldest to more recent cohorts, the numbers of differential selection signals between these two breeds would decrease in contrast to the observed results. Thus, the observed trends in the numbers of differential selection signatures may be attributed to the increased differentiation between the two breeds related to traits other than pigmentation. Alternatively, the increasingly black coat color of the more recent Podolica Italiana cattle may involve genetic mechanisms different from those responsible for the black solid coat color in the Angus breed, which could warrant further investigations.

## Conclusion

This study involves elucidating the genetic architecture of an adaptive trait (coat color) in Podolica Italiana cattle and suggests a possible role of indicine introgression in shaping the phenotypic features of this breed and perhaps other gray cattle breeds as well. The findings of this study represent a step further toward informed genetic management aimed at improving cattle resilience.

## Data Availability

The data analyzed in this study are subject to the following licenses/restrictions: Datasets are available upon request. Requests to access these datasets should be directed to Emiliano Lasagna, emiliano.lasagna@unipg.it.
