# Supplementary material for: Validation of selection signatures for coat color in the Podolica Italiana gray cattle breed
Source: Front Genet. 2024 Dec 9;15:1453295. doi: 10.3389/fgene.2024.1453295 (PMC11663911; doi:10.3389/fgene.2024.1453295)
Supplement: Supplementary file 3 [file Table8.docx]

**Supplementary file S1.** Functions of genes detected in the region on BTA7 spanning from 45,418,300 to 45,941,037 bp in ARS-UCD2.0 assembly and including two significant loci (ARS-BFGL-NGS-12557 and ARS-BFGL-NGS-20141).

***C7H5orf15:*** no relevant results.

***LOC132345820:*** no relevant results.

***VDAC1*** (Voltage Dependent Anion Channel 1) encodes a voltage-dependent anion channel protein that is a major component of the outer mitochondrial membrane, mediating the entry of metabolites (e.g., NADH, pyruvate, malate, succinate, and nucleotides) into the mitochondria and the exit of newly formed molecules such as ROS into the cytosol (Shoshan-Barmatz et al., 2018). Moreover, several studies have shown that *VDAC1* has a prominent role in maintaining intracellular $\mathrm{Ca}^{2+}$ homeostasis, mediating $\mathrm{Ca}^{2+}$ to cross mitochondrial membrane (Shoshan-Barmatz et al., 2010; Shoshan-Barmatz & Ben-Hail, 2012) and regulating the permeability of $\mathrm{Ca}^{2+}$ in the inter-mitochondrial membrane (Gincel et al., 2001; Rapizzi et al., 2002; Tan & Colombini, 2007). It has been shown that $\mathrm{Ca}^{2+}$ homeostasis is crucial for the melanogenesis (Carsberg et al., 1995; Zhang et al., 2019; Jia et al., 2020), since $\mathrm{Ca}^{2+}$ enters the melanosomes to regulate their maturation (Samuelson et al., 1993). Thus, the melanosomal-$\mathrm{Ca}^{2+}$ homeostasis is an essential process for the pigment production (Zhang et al., 2019; Le et al., 2021). In a recent study, Wang et al. (2022) demonstrated that *VDAC1* is an important negative regulator of melanogenesis in melanocytes through the Ca2+-calcineurin-CRTC1-MITF pathway. Particularly, the depletion of *VDAC1* increases pigment content and up-regulates melanogenic genes, while cultured melanocytes knockdown of *VDAC1* leads to the up-regulation of the transcription of the master regulator of melanogenesis *MITF* (Melanocyte Inducing Transcription Factor), through the activation of the Ca2+-calmodulin-CaN pathway. In the same study, the effects of *VDAC1* on pigmentation in vivo were explored by using *VDAC1*-knockout mice which showed both an increased number of melanosomes in the eyes and a decreased size of melanosomes compared to wild-type mice, thus attesting that *VDAC1* regulates melanogenesis also in vivo.

*GO*: small molecule metabolic process, transmembrane transport, symbiotic process, response to stress, cell-cell signaling, cell death, autophagy, cell differentiation, nervous system process, anatomical structure development.

***TCF7*** (Transcription Factor 7) encodes a member of the T-cell factor/lymphoid enhancer-binding factor family of high mobility group (HMG) box transcriptional activators. The encoded protein forms a complex with beta-catenin and activates transcription through a Wnt/beta-catenin signaling pathway. Wnt signals are involved in regulating the differentiation of neural crest cells into melanocytes (Patapoutian and Reichardt, 2000) through the beta-catenin associated with a nuclear mediator of the lymphoid-enhancing factor 1 (LEF-1)/T-cell factors (TCFs) family. Several studies demonstrated the involvement of different main components of the Wnt/beta-catenin signaling pathway in melanocyte expansion and differentiation (Dunn et al., 2000) or in the formation of pigment-cells of the neural crest origin (Dorsky et al., 1998).

*GO*: DNA metabolic process, reproduction, protein-containing complex assembly, cell-cell signaling, cell differentiation, signal transduction, immune system process, embryo development.

***SKP1*** (S-Phase Kinase Associated Protein 1) encodes an adaptor component of SCF (SKP1-CUL1-RBX-F-box protein) E3 ubiquitin-protein ligase complex which mediates the ubiquitination of specific protein substrates, targeting them for degradation by the proteosome. In a recent study, Liu et al. (2023) investigated the metabolic characteristics of the flavonoid pigments in the petals of *Nelumbo.* The authors demonstrated that SKP1–CUL1–F-box protein complex related genes play an important role in the biosynthesis of anthocyanins, which are the most conspicuous class of secondary plant metabolites owing to the variety of pigments that color many flowers, fruits, and seeds (Grotewold et al., 2006). Particularly, the targets for the SCF complex might be intermediate substrates of the flavonoid biosynthetic pathway or related transcription factors, and the subsequent ubiquitination and degradation of the target proteins might lead to reprogramming of the flavonoid biosynthesis pathways. The F-box protein *SKP2*, one of component of the SCF complex, is considered an oncogene involved in various malignancies (Kulinski et al., 2018; Latres et al., 2001). It has been demonstrated that this protein plays a key role in the development of uveal melanoma (UM), which originates from the neural crest-derived melanocytes in the uvea and shows different degrees of pigmentation. Notably, *SKP2* was found to be highly expressed in UM cells, promoting the progression of UM, while the targeted inhibition of *SKP2* suppresses the UM cell proliferation (Zhao et al., 2019). In the SCF complex, the N-terminal of Cullin-1 (*CUL-1*) binds the linking protein *SKP1*. A study by Voigt et Papalopulu (2005) showed that *CUL-1* is implicated in neural crest development, since it is expressed throughout early *Xenopus* development and is enriched in neural tissue. The authors demonstrated that the overexpression of a truncated dominant-negative form of *CUL-1* leads to pleiotropic defects in development, most noticeably a large increase of melanocytes, a neural crest derived cells, at the expense of cranial ganglia neurons, probably due to the inhibition of the activity of the endogenous SCF complex. Notably, blocking the function of the endogenous *CUL-1* leads to an accumulation of beta-catenin and a reduction of ubiquitinated beta-catenin, responsible for the increased expression of the early neural crest markers *Slug*, *Sox9*, *Sox10* and *Zic3*. In turn, these markers mediate the increased allocation to neural crest fate and in particular the melanocyte lineage. As an example, overexpression of beta-catenin expands *Slug* expression at the expense of the pan-neural *Sox2* (LaBonne and Bronner-Fraser, 1998) and overexpression of *Sox10* leads to a massive increase of melanocytes. Moreover, within the neural crest lineage, Wnt/beta-catenin signaling may activate genes such as the transcription factor *MITF* promoting the development of melanocytes (Yanfeng et al., 2003). Taken together, these results show how the SCF complex is involved at various levels in melanogenesis and pigmentation pathways.

*GO*: homeostatic process, cellular protein modification process, immune system process, signal transduction, cell-cell signaling, protein-containing complex assembly, symbiotic process, response to stress, chromosome organization, mitotic cell cycle, catabolic process.

***PPP2CA*** (Protein Phosphatase 2 Catalytic Subunit Alpha) encodes the phosphatase 2A catalytic subunit. Protein phosphatase 2A is one of the four major serine and threonine phosphatases, and its alpha isoform is implicated in cell proliferation, growth, metabolism and tumorigenesis. The roles of *PPP2CA* in both hair follicle and epidermis development was investigated by Fang et al. (2016), who generated conditionally knocked out *PPP2CA* mice showing, among others, visible melanin deposition and pigmentation at the base of the claws and in paws when compared to wild type mice. In mutant mice, Fang et al. (2016) found no difference regarding the total amount of *AKT* expression and a decrease of phosphorylated *AKT* (*pAKT*) in position T308, suggesting that knocking out *PPP2CA* in mice may lead to the promotion of the *AKT* pathway, known to be implicated in the beta-catenin stabilization and its nuclear accumulation. The latter is the central substrate in Wnt signaling (Lee et al., 2009) which is closely related to melanocyte development and melanogenesis, ending with the upregulation of *MITF* transcription and the expression of genes involved in melanogenesis (Liu et al., 2020; Molagoda et al., 2020; Zang et al., 2019). Nevertheless, these results are not completely consistent with those reported by Pandey et al. (2013) which demonstrated that the downregulation of *PPP2CA* leads to an increase of *pAKT*, but in position S473, activating the same above-mentioned pathways.

*GO*: membrane organization, nucleoblase-containing compound catabolic process, lipid metabolic process, cellular protein modification process, signal transduction, mitotic cell cycle, reproduction, anatomical structure development, cell death.

***LOC112447418***: no relevant results.

***MIR2285DI*** encodes the *Bos taurus* bta-mir-2285di precursor miRNA. Melanogenesis is subject to several external and internal stimuli which operate through different intracellular signal pathways, finally leading to the regulation of *MITF* and to the differential expression of the main melanogenic enzymes (*TYR*, *TYRP-1*, and *TYRP-2*). Among the internal factors influencing melanogenesis there are also several epigenetic factors, including microRNAs (miRNAs) (Horsburgh et al., 2017; Yamada et al., 2019; Jiang et al., 2020; Lanzillotti et al., 2021). miRNA activity regulates gene expression mainly by binding the 3′-UTR of target genes, thus leading to mRNA target degradation or inhibition of translation and reduction in protein levels (Mazziotta et al., 2021). Several studies have been conducted to investigate the effects of miRNAs on melanogenesis. In different coat colored fiber-producing animals, such as *Vicugna pacos*, differences in miRNA profiles were found and most differentially expressed miRNAs showed predicted targets involved in pigmentation (Zhu et al., 2010; Tian et al., 2012). In 2012 the role of miR-137, a miRNA targeting *MITF*, in a transgenic mice model was investigated (Dong et al., 2012). The authors showed that miR-137 decreased the expression of the *MITF* protein and its downstream genes, having also an impact on coat color of transgenic mice. To our current knowledge, to date a total of 11 published articles mentioned *MIR2285DI* in *Bos taurus*, even if not associated to melanogenesis or pigmentation pathways.

***CDKL3:*** no relevant results.

*GO*: growth, cell morphogenesis, cellular protein modification process, cell differentiation.

**REFERENCES**

Carsberg, C. J., Jones, K. T., Sharpe, G. R., and Friedmann, P. S. (1995). Intracellular calcium modulates the responses of human melanocytes to melanogenic stimuli. *J Dermatol Sci* 9, 157–164. doi: [10.1016/0923-1811(94)00372-l](https://doi.org/10.1016/0923-1811(94)00372-l)

Dong, C., Wang, H., Xue, L., Dong, Y., Yang, L., Fan, R., et al. (2012). Coat color determination by miR-137 mediated down-regulation of microphthalmia-associated transcription factor in a mouse model. *RNA* 18, 1679–1686. doi: 10.1261/rna.033977.112

Dorsky, R. I., Moon, R. T., and Raible, D. W. (1998). Control of neural crest cell fate by the Wnt signalling pathway. *Nature* 396, 370–373. doi: [10.1038/24620](https://doi.org/10.1038/24620)

Dunn, K. J., Williams, B. O., Li, Y., and Pavan, W. J. (2000). Neural crest-directed gene transfer demonstrates Wnt1 role in melanocyte expansion and differentiation during mouse development. *Proc Natl Acad Sci U S A* 97, 10050–10055.

Fang, C., Li, L., and Li, J. (2016). Conditional Knockout in Mice Reveals the Critical Roles of Ppp2ca in Epidermis Development. *Int J Mol Sci* 17, 756. doi: [10.3390/ijms17050756](https://doi.org/10.3390/ijms17050756)

Gincel, D., Zaid, H., and Shoshan-Barmatz, V. (2001). Calcium binding and translocation by the voltage-dependent anion channel: a possible regulatory mechanism in mitochondrial function. *Biochem J* 358, 147–155.

Grotewold, E. (2006). The genetics and biochemistry of floral pigments. *Annu Rev Plant Biol* 57, 761–780. doi: [10.1146/annurev.arplant.57.032905.105248](https://doi.org/10.1146/annurev.arplant.57.032905.105248)

Horsburgh, S., Fullard, N., Roger, M., Degnan, A., Todryk, S., Przyborski, S., et al. (2017). MicroRNAs in the skin: role in development, homoeostasis and regeneration. *Clin Sci (Lond)* 131, 1923–1940. doi: 10.1042/CS20170039

Jia, Q., Hu, S., Jiao, D., Li, X., Qi, S., and Fan, R. (2020). Synaptotagmin-4 promotes dendrite extension and melanogenesis in alpaca melanocytes by regulating Ca2+ influx via TRPM1 channels. *Cell Biochem Funct* 38, 275–282. doi: [10.1002/cbf.3465](https://doi.org/10.1002/cbf.3465)

Jiang, L., Huang, J., Hu, Y., Lei, L., Ouyang, Y., Long, Y., et al. (2020). Identification of the ceRNA networks in α-MSH-induced melanogenesis of melanocytes. *Aging (Albany NY)* 13, 2700–2726. doi: 10.18632/aging.202320

Kulinski, M., Achkar, I. W., Haris, M., Dermime, S., Mohammad, R. M., and Uddin, S. (2018). Dysregulated expression of SKP2 and its role in hematological malignancies. *Leuk Lymphoma* 59, 1051–1063. doi: [10.1080/10428194.2017.1359740](https://doi.org/10.1080/10428194.2017.1359740)

LaBonne, C., and Bronner-Fraser, M. (1998). Neural crest induction in Xenopus: evidence for a two-signal model. *Development* 125, 2403–2414. doi: [10.1242/dev.125.13.2403](https://doi.org/10.1242/dev.125.13.2403)

Lanzillotti, C., De Mattei, M., Mazziotta, C., Taraballi, F., Rotondo, J. C., Tognon, M., et al. (2021). Long Non-coding RNAs and MicroRNAs Interplay in Osteogenic Differentiation of Mesenchymal Stem Cells. *Front Cell Dev Biol* 9, 646032. doi: 10.3389/fcell.2021.646032

Latres, E., Chiarle, R., Schulman, B. A., Pavletich, N. P., Pellicer, A., Inghirami, G., et al. (2001). Role of the F-box protein Skp2 in lymphomagenesis. *Proc Natl Acad Sci U S A* 98, 2515–2520. doi: [10.1073/pnas.041475098](https://doi.org/10.1073/pnas.041475098)

Le, L., Sirés-Campos, J., Raposo, G., Delevoye, C., and Marks, M. S. (2021). Melanosome Biogenesis in the Pigmentation of Mammalian Skin. *Integr Comp Biol* 61, 1517–1545. doi: [10.1093/icb/icab078](https://doi.org/10.1093/icb/icab078)

Lee, J.-Y., Kang, M.-B., Jang, S.-H., Qian, T., Kim, H.-J., Kim, C.-H., et al. (2009). Id-1 activates Akt-mediated Wnt signaling and p27(Kip1) phosphorylation through PTEN inhibition. *Oncogene* 28, 824–831. doi: [10.1038/onc.2008.451](https://doi.org/10.1038/onc.2008.451)

Liu, B., Xie, Y., and Wu, Z. (2020). Astragaloside IV Enhances Melanogenesis via the AhR-Dependent AKT/GSK-3β/β-Catenin Pathway in Normal Human Epidermal Melanocytes. *Evid Based Complement Alternat Med* 2020, 8838656. doi: [10.1155/2020/8838656](https://doi.org/10.1155/2020/8838656)

Liu, X., Du, F., Sun, L., Li, J., Chen, S., Li, N., et al. (2023). Anthocyanin metabolism in Nelumbo: translational and post-translational regulation control transcription. *BMC Plant Biol* 23, 61. doi: [10.1186/s12870-023-04068-3](https://doi.org/10.1186/s12870-023-04068-3)

Mazziotta, C., Lanzillotti, C., Iaquinta, M.R., Taraballi, F., Torreggiani, E., Rotondo, J.C., et al. (2021). MicroRNAs Modulate Signaling Pathways in Osteogenic Differentiation of Mesenchymal Stem Cells. *Int J Mol Sci* 2021, 22, 2362. https://doi.org/10.3390/ ijms22052362

Molagoda, I. M. N., Karunarathne, W. A. H. M., Park, S. R., Choi, Y. H., Park, E. K., Jin, C.-Y., et al. (2020). GSK-3β-Targeting Fisetin Promotes Melanogenesis in B16F10 Melanoma Cells and Zebrafish Larvae through β-Catenin Activation. *Int J Mol Sci* 21, 312. doi: [10.3390/ijms21010312](https://doi.org/10.3390/ijms21010312)

Pandey, P., Seshacharyulu, P., Das, S., Rachagani, S., Ponnusamy, M. P., Yan, Y., et al. (2013). Impaired expression of protein phosphatase 2A subunits enhances metastatic potential of human prostate cancer cells through activation of AKT pathway. *Br J Cancer* 108, 2590–2600. doi: [10.1038/bjc.2013.160](https://doi.org/10.1038/bjc.2013.160)

Patapoutian, A., and Reichardt, L. F. (2000). Roles of Wnt proteins in neural development and maintenance. *Curr Opin Neurobiol* 10, 392–399.

Rapizzi, E., Pinton, P., Szabadkai, G., Wieckowski, M. R., Vandecasteele, G., Baird, G., et al. (2002). Recombinant expression of the voltage-dependent anion channel enhances the transfer of Ca2+ microdomains to mitochondria. *J Cell Biol* 159, 613–624. doi: [10.1083/jcb.200205091](https://doi.org/10.1083/jcb.200205091)

Samuelson, D. A., Smith, P., Ulshafer, R. J., Hendricks, D. G., Whitley, R. D., Hendricks, H., et al. (1993). X-ray Microanalysis of Ocular Melanin in Pigs Maintained on Normal and Low Zinc Diets. *Experimental Eye Research* 56, 63–70. doi: [10.1006/exer.1993.1009](https://doi.org/10.1006/exer.1993.1009)

Shoshan-Barmatz, V., and Ben-Hail, D. (2012). VDAC, a multi-functional mitochondrial protein as a pharmacological target. *Mitochondrion* 12, 24–34. doi: [10.1016/j.mito.2011.04.001](https://doi.org/10.1016/j.mito.2011.04.001)

Shoshan-Barmatz, V., De Pinto, V., Zweckstetter, M., Raviv, Z., Keinan, N., and Arbel, N. (2010). VDAC, a multi-functional mitochondrial protein regulating cell life and death. *Mol Aspects Med* 31, 227–285. doi: [10.1016/j.mam.2010.03.002](https://doi.org/10.1016/j.mam.2010.03.002)

Shoshan-Barmatz, V., Nahon-Crystal, E., Shteinfer-Kuzmine, A., and Gupta, R. (2018). VDAC1, mitochondrial dysfunction, and Alzheimer’s disease. *Pharmacol Res* 131, 87–101. doi: [10.1016/j.phrs.2018.03.010](https://doi.org/10.1016/j.phrs.2018.03.010)

Tan, W., and Colombini, M. (2007). VDAC closure increases calcium ion flux. *Biochim Biophys Acta* 1768, 2510–2515. doi: [10.1016/j.bbamem.2007.06.002](https://doi.org/10.1016/j.bbamem.2007.06.002)

Tian, X., Jiang, J., Fan, R., Wang, H., Meng, X., He, X., et al. (2012). Identification and characterization of microRNAs in white and brown alpaca skin. *BMC Genomics* 13, 555. doi: 10.1186/1471-2164-13-555

Voigt, J., and Papalopulu, N. (2006). A dominant-negative form of the E3 ubiquitin ligase Cullin-1 disrupts the correct allocation of cell fate in the neural crest lineage. *Development* 133, 559–568. doi: [10.1242/dev.02201](https://doi.org/10.1242/dev.02201)

Wang, J., Gong, J., Wang, Q., Tang, T., and Li, W. (2022). VDAC1 negatively regulates melanogenesis through the Ca2+-calcineurin-CRTC1-MITF pathway. *Life Sci Alliance* 5, e202101350. doi: [10.26508/lsa.202101350](https://doi.org/10.26508/lsa.202101350)

Yamada, T., Hasegawa, S., Iwata, Y., Arima, M., Kobayashi, T., Numata, S., et al. (2019). UV irradiation-induced DNA hypomethylation around WNT1 gene: Implications for solar lentigines. *Exp Dermatol* 28, 723–729. doi: 10.1111/exd.13949

Yanfeng, W., Saint-Jeannet, J.-P., and Klein, P. S. (2003). Wnt-frizzled signaling in the induction and differentiation of the neural crest. *Bioessays* 25, 317–325. doi: [10.1002/bies.10255](https://doi.org/10.1002/bies.10255)

Zang, D., Niu, C., and Aisa, H. A. (2019). Amine derivatives of furocoumarin induce melanogenesis by activating Akt/GSK-3β/β-catenin signal pathway. *Drug Des Devel Ther* 13, 623–632. doi: [10.2147/DDDT.S180960](https://doi.org/10.2147/DDDT.S180960)

Zhang, Z., Gong, J., Sviderskaya, E. V., Wei, A., and Li, W. (2019). Mitochondrial NCKX5 regulates melanosomal biogenesis and pigment production. *J Cell Sci* 132, jcs232009. doi: [10.1242/jcs.232009](https://doi.org/10.1242/jcs.232009)

Zhao, H., Pan, H., Wang, H., Chai, P., Ge, S., Jia, R., et al. (2019). SKP2 targeted inhibition suppresses human uveal melanoma progression by blocking ubiquitylation of p27. *Onco Targets Ther* 12, 4297–4308. doi: [10.2147/OTT.S203888](https://doi.org/10.2147/OTT.S203888)

Zhu, Z., He, J., Jia, X., Jiang, J., Bai, R., Yu, X., et al. (2010). MicroRNA-25 functions in regulation of pigmentation by targeting the transcription factor MITF in Alpaca (Lama pacos) skin melanocytes. *Domest Anim Endocrinol* 38, 200–209. doi: 10.1016/j.domaniend.2009.10.004
