# Supplementary material for: Validation of selection signatures for coat color in the Podolica Italiana gray cattle breed
Source: Front Genet. 2024 Dec 9;15:1453295. doi: 10.3389/fgene.2024.1453295 (PMC11663911; doi:10.3389/fgene.2024.1453295)
Supplement: Supplementary file 4 [file Table9.docx]

**Supplementary file S2.** Functions of genes detected in the region on BTA18 spanning from 13,819,857 to 14,607,607 bp in ARS-UCD2.0 assembly and including three significant loci (ARS-BFGL-NGS-4996, ARS-BFGL-NGS-31386 and Hapmap44238-BTA-42338).

***LOC517901:*** no relevant results.

***ZC3H18:*** no relevant results.

***CYBA*** (Cytochrome B-245 Alpha Chain) encodes the protein p22phox, that is a critical component of the superoxide-generating NADPH oxidases (NOXs). This protein is associated with NOX2 to form the redox element cytochrome b expressed mainly in phagocytes and generating ROS starting from O2. Notably, cytochrome b, is a membrane heterodimer composed of two subunits: p22phox (also called alpha subunit or light chain) and NOX2 (also named beta subunit or heavy chain and encoded by *CYBB*). Oxidative stress and mitochondrial dysfunction, characterized by elevated ROS and mtROS levels, are crucial for age-related diseases, especially those involving the amyloid β (Aβ)-induced neuron degeneration, such as Alzheimer’s disease and age-related macular degeneration (AMD) (Lashkari et al., 2018). In a recent study, Sun et al., (2020) demonstrated that the Aβ deposition, an essential pathological process in AMD, causes oxidative stress leading to the retina pigment epithelium (RPE) degeneration. Notably, the authors identified several subunits of the NADPH oxidase (i.e. *CYBA*, *CYBB*, *NOX4*, *NCF2* and *NCF4*) as hub proteins in Aβ-mediated oxidative stress, since they were find to be upregulated in RPE cells after exposure to Aβ.

In human and mice, the synthesis of melanin by melanocytes is mainly regulated by the alpha-melanocyte stimulating hormones (α-*MSH*) that binds and activates the melanocortin 1 receptor (*MC-1R*), thus activating the cAMP pathway (Yamaguchi et al., 2007; Abdel-Malek et al., 2000; Garcia-Borron et al., 2005). The latter, once activated, triggers its downstream effectors in order to up-regulate the expression of *MITF* (Bertolotto et al., 1998), leading to melanocyte differentiation. Therefore, α-MSH/MC-1R/MITF signaling appears to be fundamental in melanogenesis and pigmentation. With reference to the above-described mechanism, in another study, Liu et al., (2012) showed how NADPH oxidase-derived ROS modulates the expression of the pigment “melanin” synthesizing enzymes in mouse melanoma cells. In particular, they demonstrated that α-*MSH* enhanced both the expression of NADPH oxidase 4 (Nox4) and ROS generation in cells. Moreover, silencing Nox4 by using Nox4 siRNA leads an increase of melanin development, demonstrating that constitutive or stimulated Nox4-dependent ROS inhibits melanin formation. Taken together, these results suggest that α-MSH/MC-1R/MITF signaling stimulates Nox4 to drive ROS generation, thereby repressing melanin synthesis.

*GO*: homeostatic process, circulatory system process, generation of precursor metabolites and energy, signal transduction, immune system process, cofactor metabolic process, vesicle-mediated transport, biosynthetic process, response to stress, protein-containing complex assembly.

***MVD:*** no relevant results.

*GO*: lipid metabolic process, sulfur compound metabolic process, small molecule metabolic process, cellular nitrogen compound metabolic process, cellular protein modification process, biosynthetic process, cofactor metabolic process.

***LOC132342792:*** no relevant results.

***SNAI3:*** no relevant results.

***CTU2:*** no relevant results.

*GO*: cellular protein modification process, tRNA metabolic process.

***RNF166:*** no relevant results.

***PIEZO1:*** no relevant results.

*GO*: transmembrane transport.

***MIR2327:*** no relevant results.

***CDT1:*** no relevant results.

*GO*: biosynthetic process, protein-containing complex assembly, mitotic nuclear division, cell division, chromosome segregation, chromosome organization.

***APRT:*** no relevant results.

*GO*: immune system process, small molecule metabolic process, cellular nitrogen compound metabolic process, vesicle-mediated transport, anatomical structure development, biosynthetic process.

***GALNS:*** no relevant results.

*GO*: immune system process, catabolic process, small molecule metabolic process, sulfur compound metabolic process, vesicle-mediated transport.

***TRAPPC2L:*** no relevant results.

***PABPN1L:*** no relevant results.

***CBFA2T3:*** no relevant results.

***LOC132342793:*** no relevant results.

***ACSF3*** (Acyl-CoA Synthetase Family Member 3) encodes a member of the acyl-CoA synthetase family of enzymes, involved in fatty acid biosynthesis, that catalyze reaction between malonate and coenzyme A (CoA), generating the malonyl-CoA. This product is one of the main precursors for synthesizing and elongating fatty acids. Additionally, it serves as a substrate for the CHS enzyme (the first enzyme in the flavonoid and isoflavonoid formation pathway) during the synthesis of flavonoids and anthocyanins (Chen et al., 2011; Dastmalchi et al., 2015), which are plant metabolites owing to the variety of pigments given color to many flowers, fruits, and seeds (Grotewold et al., 2006). A recent study contrasting the transcriptional profile of different coat color soybean seed (yellow *vs* black) showed that *ACSF3* was 4× more expressed in the black seed coat, suggesting its role in black pigmentation pathways of soybean seeds (Kafer et al.,2023).

*GO*: biosynthetic process, lipid metabolic process, catabolic process, cofactor metabolic process, cellular nitrogen compound metabolic process, small molecule metabolic process, sulfur compound metabolic process.

***CDH15*** (Cadherin 15) is a member of the cadherin superfamily of genes, encoding calcium-dependent intercellular adhesion glycoproteins. Even if a selective sweep study in cattle populations identified *CDH15* as candidates for meat production (Gutiérrez-Gil et al., 2015), more recently a similar analysis performed on Hainan Black Goat associated this gene with regulation of melanin biosynthetic process (Chen et al., 2022). Notably, *CDH15* resulted to be one of the selective signature genes between the Hainan Black Goat and the whiteness-known Alashan Cashmere goat by both the gene ontology analysis, based on biological processes, and enrichment analysis performed.

*GO*: cellular component assembly, cell junction organization, cell adhesion, cell morphogenesis.

***SLC22A31***: no relevant results.

*GO*: transmembrane transport.

***ANKRD11*** (Ankyrin Repeat Domain Containing 11) encodes an ankryin repeat domain-containing protein that inhibits ligand-dependent activation of transcription. This gene is a chromatin regulator, suggesting that its decrease in expression could impact the transcriptional levels of additional genes downstream. It has been shown that mice with homozygous neural crest-specific deletion of *ANKRD11* result in severe craniofacial phenotypes, including the loss of black pigment on the nose (Roth et al., 2021). Another study by He et al. (2022), investigating the hereditary basis of coat color in Red Angus cattle, identified some SNPs in *ANKRD11* gene considerably diverged between Black and Red Angus cattle. Although no direct evidence showed that *ANKRD11* is related to oxidative phosphorylation (MOP), it interacts with a large number of genes which participate in the process of MOP (Rao et al., 2019; Song et al., 2017). Since the MOP is a cellular process generating energy in the form of adenosine triphosphate (ATP) and ROS as byproducts (Deshpande et al., 2024), it could lead to oxidative stress (OS) which is highly involved in melanin synthesis and melanoma development. Notably, the increased production of ROS activates the ROS-ERK signaling pathway, leading to phosphorylation and proteasomal degradation of MITF and downregulation of melanogenesis (Kim et al., 2018).

*GO*: embryo development, homeostatic process, growth.

***LOC132342794***: no relevant results.

***LOC112442271***: no relevant results.

***SPG7***: no relevant results.

*GO*: mitochondrion organization, anatomical structure development, cell death, membrane organization, cytoskeleton-dependent intracellular transport, transmembrane transport.

***RPL13*** (Ribosomal Protein L13) encodes a ribosomal protein that is a component of the large 60S subunit of ribosomes. Ribosomes are the organelles catalyzing protein synthesis. They are organized into an 80S structure consisting of a small 40S subunit and a large 60S subunit (Ben-Shem et al., 2011). Kardos et al., (2014) investigated the role of the large 60S subunit ribosomal proteins (RPLs) in melanoma and categorized RPLs based on modulation of cell proliferation in two groups: the first causing negligible effects on cell viability and the second causing a decrease in cell viability. Interestingly, the authors demonstrated that *RPL13* belong to the second category, since they found that *RPL13* was expressed in melanocytes, and advanced stage melanoma cell lines had four to six times greater *RPL13* protein expression, suggesting it was important for advanced melanoma cell survival. In addition, siRNA targeting of *RPL13* decreased melanoma cell proliferation and viability through the MDM2-p53 pathway. Notably, decreased *RPL13* levels increased p53 stability by preventing MDM2 (Mouse Double Minute 2, an oncoprotein) from targeting p53 (a tumor suppressor) for degradation, leading to a p53-dependent cell cycle arrest.

*GO*: translation, nucleobase-containing compound catabolic process, symbiotic process, protein targeting.

***LOC112442426***: no relevant results.

***CPNE7***: no relevant results.

*GO*: lipid metabolic process, biosynthetic process.

***DPEP1***: no relevant results.

*GO*: cell death, catabolic process, response to stress, cofactor metabolic process, sulfur compound metabolic process, cellular nitrogen compound metabolic process, cellular amino acid metabolic process.

***CHMP1A*** (Charged Multivesicular Body Protein 1A) encodes a member of the CHMP/Chmp family of proteins which are involved in multivesicular body sorting of proteins to the interiors of lysosomes. According to the GWAS Catalog (https://www.ebi.ac.uk/gwas/home), the *CHMP1A* gene was associated with the traits of hair color, hair color measurement, skin pigmentation measurement and skin sensitivity to sun in human. Notably, Farré et al. (2023) reported that an intronic deletion of *CHMP1A* gene, located close to *MC1R*, was significantly associated with red hair.

*GO*: protein transport, vacuolar transport, cell division, chromosome segregation, mitotic nuclear division, cellular component assembly, chromosome organization, vescicle-mediated transport, symbiotic process, membrane organization.

***SPATA33*** (Spermatogenesis Associated 3) encodes a protein playing an important role in sperm motility and male fertility. In addition, links damaged mitochondria to autophagosomes via its binding to the outer mitochondrial membrane protein *VDAC2*, as well as to key autophagy machinery component *ATG16L1*. *SPATA33* was identified in a functional annotation study of melanoma risk as putative melanoma susceptibility gene (Fang et al., 2020). Moreover, it has been associated with Fanconi Anemia, a rare genetic disorder characterized by widespread areas of hyper- and hypopigmentation of the skin and café-au-lait macules (Ruggiero et al., 2021; Ogilvie et al., 2002). The latter could be explained by the association of *SPATA33* gene with facial pigmentation spots (Jacobs et al., 2015) and/or with skin, red hair and phototype, i.e. a skin classification not only defined by the skin color, but also including other pigmentary traits such as hair and eye color, presence of freckles and sunlight sensitivity (Farré et al., 2023).

***CDK10*** (Cyclin Dependent Kinase 10) encodes for a protein belonging to the CDK subfamily of the Ser/Thr protein kinase family. This gene regulates cell cycle progression from the G2 to the M phase (Li et al., 1995). As reported in GWAS Catalog (https://www.ebi.ac.uk/gwas/home), several GWAS studies associated *CDK10* to the traits of hair color and hair color measurement in human. In a study by Lona-Durazo et al. (2021) a GWAS meta-analysis of hair color in a Canadian cohort of 12,741 individuals with European ancestry was conducted in order to identify candidate casual variants in pigmentation loci associated with blonde, red and brown hair color. The results of this study demonstrated that *CDK10* was significantly associated with hair color. Notably, a SNP within *CDK10* was a colocalizing methylation quantitative trait locus (meQTL) for blonde hair color, while the transcriptome-wide association study (TWAS) demonstrated that the decreased expression of *CDK10* was significantly associated with red hair color. In addition, an omics analysis identified *CDK10* as a putative relevant gene associated with vitiligo, a multifactorial polygenic disorder, characterized by acquired depigmented skin and overlying hair resulting from the destruction of melanocytes (Cai et al., 2021). The association was validated by qPCR, showing that *CDK10* was significantly upregulated in the blood of vitiligo patients compared to healthy controls (Cai et al., 2021). Since *CDK10* is considered a key candidate tumor suppressor in several cancer types (Zhong et al., 2012; Ransohoff et al., 2017; You et al., 2018), these results supported the evidence that patients with vitiligo have a reduced risk of malignancies (Bae et al., 2019), melanoma, and non-melanoma skin cancer (Paradisi et al., 2014).

*GO*: cellular protein modification process.

***LOC104974758***: no relevant results.

***SPATA2L*** (Spermatogenesis Associated 2 Like) encodes the paralog of *SPATA2* that is involved in several processes, including protein deubiquitination, regulation of necroptotic process and regulation of tumor necrosis factor-mediated signaling pathway. According to several studies, *SPATA2L* is associated to hair color and hair color measurement traits, as also reported in the GWAS Catalog. As an example, Cai et al. (2021) identified *SPATA2L* as a newly putative functional gene associated with vitiligo susceptibility, by performing the Summary data-based Mendelian Randomization (SMR) and the Heterogeneity in Dependent Instruments (HEIDI) tests. Another study by Bonilla et al. (2021), investigating DNA methylation as a potential mediator between pigmentation genes, pigmentary traits and skin cancer, demonstrated that *SPATA2L* showed a potential pleiotropic association with the phenotypes of skin color and black hair, since these traits colocalized with the DNAm site near *MC1R* (Melanocortin 1 Receptor) gene, known to be responsible of coat color variation in cattle (Goud et al., 2021).

***VPS9D1***: no relevant results.

*GO*: biosynthetic process, cellular nitrogen compound metabolic process, small molecule metabolic process, transmembrane transport.

***ZNF276*** (Zinc Finger Protein 276) encodes a protein that is predicted to be involved in regulation of transcription by RNA polymerase II. The GWAS Catalog (https://www.ebi.ac.uk/gwas/home) reported that it is associated with several traits, among which hair color, hair color measurement and skin pigmentation measurement resulted to be interesting. In a study by Zhang et al. (2020), investigating the selection signatures in a Chinese native pig (Anqing six-end-white pig) characterized by black coat color, *ZNF276* gene was found to be a promising candidate gene that affects the metabolism and cell process. In addition, Farrè et al. (2023) characterized the functional impact of the SNPs identified with a GWAS approach by using a gene-based analysis and, subsequently, tested the association between genes and pigmentary traits identifying an association of *ZNF276* with red hair in human.

***FANCA*** (Fanconi Anemia Complementation Group A) encodes a member of the Fanconi Anemia Complementation group (FANC), which currently includes *FANCA, FANCB, FANCC, FANCD1* (also called *BRCA2*), *FANCD2, FANCE, FANCF, FANCG, FANCI, FANCJ* (also called *BRIP1*), *FANCL, FANCM* and *FANCN* (also called *PALB2*). The member of the FANC do not share sequence similarity, but they are related by their assembly into a common nuclear protein complex. *FANCA* encodes the protein for the Complementation group A. Mutation in this gene are the most common cause of Fanconi anemia (George et al., 2021; Bagby, 2018; Chandrasekharappa et al., 2013; Madjunkova et al., 2014), a rare genetically heterogeneous recessive disorder associated with variable clinical manifestations. The latter include cutaneous pigmentary alterations such as widespread areas of hyper- and hypopigmentation of the skin in a characteristic pattern and café-au-lait spots (Ruggiero et al., 2021; Ogilvie et al., 2002). In Red *vs* Black Angus cattle, the hereditary basis of coat color was investigated by He et al. (2022), who used a general linear model analysis to identify genomic regions with potential candidate variant/genes that contribute to the considered phenotype. Results showed that three consecutives strongly associated SNPs, identified and annotated in *FANCA*, considerably diverged between Black and Red Angus cattle.

*GO*: cell cycle, DNA metabolic process, anatomical structure development, response to stress, protein-containing complex assembly, reproduction.

**REFERENCES**

Abdel-Malek, Z., Scott, M. C., Suzuki, I., Tada, A., Im, S., Lamoreux, L., et al. (2000). The melanocortin-1 receptor is a key regulator of human cutaneous pigmentation. *Pigment Cell Res* 13 Suppl 8, 156–162. doi: [10.1034/j.1600-0749.13.s8.28.x](https://doi.org/10.1034/j.1600-0749.13.s8.28.x)

Bae, J. M., Chung, K. Y., Yun, S. J., Kim, H., Park, B. C., Kim, J. S., et al. (2019). Markedly Reduced Risk of Internal Malignancies in Patients With Vitiligo: A Nationwide Population-Based Cohort Study. *J Clin Oncol* 37, 903–911. doi: [10.1200/JCO.18.01223](https://doi.org/10.1200/JCO.18.01223)

Bagby, G. (2018). Recent advances in understanding hematopoiesis in Fanconi Anemia. *F1000Res* 7, 105. doi: [10.12688/f1000research.13213.1](https://doi.org/10.12688/f1000research.13213.1)

Ben-Shem, A., Garreau de Loubresse, N., Melnikov, S., Jenner, L., Yusupova, G., and Yusupov, M. (2011). The structure of the eukaryotic ribosome at 3.0 Å resolution. *Science* 334, 1524–1529. doi: [10.1126/science.1212642](https://doi.org/10.1126/science.1212642)

Bertolotto, C., Abbe, P., Hemesath, T. J., Bille, K., Fisher, D. E., Ortonne, J. P., et al. (1998). Microphthalmia gene product as a signal transducer in cAMP-induced differentiation of melanocytes. *J Cell Biol* 142, 827–835. doi: [10.1083/jcb.142.3.827](https://doi.org/10.1083/jcb.142.3.827)

Bonilla, C., Bertoni, B., Min, J. L., Hemani, G., and Elliott, H. R. (2021). Investigating DNA methylation as a potential mediator between pigmentation genes, pigmentary traits and skin cancer. *Pigment Cell Melanoma Res* 34, 892–904. doi: [10.1111/pcmr.12948](https://doi.org/10.1111/pcmr.12948)

Cai, M., Yuan, T., Huang, H., Gui, L., Zhang, L., Meng, Z., et al. (2021). Integrative Analysis of Omics Data Reveals Regulatory Network of CDK10 in Vitiligo Risk. *Front Genet* 12, 634553. doi: [10.3389/fgene.2021.634553](https://doi.org/10.3389/fgene.2021.634553)

Chandrasekharappa, S. C., Lach, F. P., Kimble, D. C., Kamat, A., Teer, J. K., Donovan, F. X., et al. (2013). Massively parallel sequencing, aCGH, and RNA-Seq technologies provide a comprehensive molecular diagnosis of Fanconi anemia. *Blood* 121, e138-148. doi: [10.1182/blood-2012-12-474585](https://doi.org/10.1182/blood-2012-12-474585)

Chen, H., Kim, H. U., Weng, H., and Browse, J. (2011). Malonyl-CoA Synthetase, Encoded by ACYL ACTIVATING ENZYME13, Is Essential for Growth and Development of Arabidopsis[C][W][OA]. *Plant Cell* 23, 2247–2262. doi: [10.1105/tpc.111.086140](https://doi.org/10.1105/tpc.111.086140)

Chen, Q., Chai, Y., Zhang, W., Cheng, Y., Zhang, Z., An, Q., et al. (2022). Whole-Genome Sequencing Reveals the Genomic Characteristics and Selection Signatures of Hainan Black Goat. *Genes (Basel)* 13, 1539. doi: [10.3390/genes13091539](https://doi.org/10.3390/genes13091539)

Dastmalchi, M., and Dhaubhadel, S. (2015). Proteomic insights into synthesis of isoflavonoids in soybean seeds. *Proteomics* 15, 1646–1657. doi: [10.1002/pmic.201400444](https://doi.org/10.1002/pmic.201400444)

Deshpande, O. A., and Mohiuddin, S. S. (2024). “Biochemistry, Oxidative Phosphorylation,” in *StatPearls*, (Treasure Island (FL): StatPearls Publishing). Available at: <http://www.ncbi.nlm.nih.gov/books/NBK553192/>

Fang, S., Lu, J., Zhou, X., Wang, Y., Ross, M. I., Gershenwald, J. E., et al. (2020). Functional annotation of melanoma risk loci identifies novel susceptibility genes. *Carcinogenesis* 41, 452–457. doi: [10.1093/carcin/bgz173](https://doi.org/10.1093/carcin/bgz173)

Farré, X., Blay, N., Cortés, B., Carreras, A., Iraola-Guzmán, S., and de Cid, R. (2023). Skin Phototype and Disease: A Comprehensive Genetic Approach to Pigmentary Traits Pleiotropy Using PRS in the GCAT Cohort. *Genes (Basel)* 14, 149. doi: [10.3390/genes14010149](https://doi.org/10.3390/genes14010149)

García-Borrón, J. C., Sánchez-Laorden, B. L., and Jiménez-Cervantes, C. (2005). Melanocortin-1 receptor structure and functional regulation. *Pigment Cell Res* 18, 393–410. doi: [10.1111/j.1600-0749.2005.00278.x](https://doi.org/10.1111/j.1600-0749.2005.00278.x)

George, M., Solanki, A., Chavan, N., Rajendran, A., Raj, R., Mohan, S., et al. (2021). A comprehensive molecular study identified 12 complementation groups with 56 novel FANC gene variants in Indian Fanconi anemia subjects. *Hum Mutat* 42, 1648–1665. doi: [10.1002/humu.24286](https://doi.org/10.1002/humu.24286)

Goud, T. S., Upadhyay, R. C., Pichili, V. B. R., Onteru, S. K., and Chadipiralla, K. (2021). Molecular characterization of coat color gene in Sahiwal versus Karan Fries bovine. *J Genet Eng Biotechnol* 19, 22. doi: [10.1186/s43141-021-00117-2](https://doi.org/10.1186/s43141-021-00117-2)

Grotewold, E. (2006). The genetics and biochemistry of floral pigments. *Annu Rev Plant Biol* 57, 761–780. doi: [10.1146/annurev.arplant.57.032905.105248](https://doi.org/10.1146/annurev.arplant.57.032905.105248)

Gutiérrez-Gil, B., Arranz, J. J., and Wiener, P. (2015). An interpretive review of selective sweep studies in Bos taurus cattle populations: identification of unique and shared selection signals across breeds. *Front Genet* 6, 167. doi: [10.3389/fgene.2015.00167](https://doi.org/10.3389/fgene.2015.00167)

He, Y., Huang, Y., Wang, S., Zhang, L., Gao, H., Zhao, Y., et al. (2022). Hereditary Basis of Coat Color and Excellent Feed Conversion Rate of Red Angus Cattle by Next-Generation Sequencing Data. *Animals* 12, 1509. doi: [10.3390/ani12121509](https://doi.org/10.3390/ani12121509)

Jacobs, L. C., Hamer, M. A., Gunn, D. A., Deelen, J., Lall, J. S., van Heemst, D., et al. (2015). A Genome-Wide Association Study Identifies the Skin Color Genes IRF4, MC1R, ASIP, and BNC2 Influencing Facial Pigmented Spots. *J Invest Dermatol* 135, 1735–1742. doi: [10.1038/jid.2015.62](https://doi.org/10.1038/jid.2015.62)

Kafer, J. M., Molinari, M. D. C., Henning, F. A., Koltun, A., Marques, V. V., Marin, S. R. R., et al. (2023). Transcriptional Profile of Soybean Seeds with Contrasting Seed Coat Color. *Plants (Basel)* 12, 1555. doi: [10.3390/plants12071555](https://doi.org/10.3390/plants12071555)

Kardos, G. R., Dai, M.-S., and Robertson, G. P. (2014). Growth inhibitory effects of large subunit ribosomal proteins in melanoma. *Pigment Cell & Melanoma Research* 27, 801–812. doi: [10.1111/pcmr.12259](https://doi.org/10.1111/pcmr.12259)

Kim, H.-Y., Sah, S. K., Choi, S. S., and Kim, T.-Y. (2018). Inhibitory effects of extracellular superoxide dismutase on ultraviolet B-induced melanogenesis in murine skin and melanocytes. *Life Sci* 210, 201–208. doi: [10.1016/j.lfs.2018.08.056](https://doi.org/10.1016/j.lfs.2018.08.056)

Lashkari, K., Teague, G., Chen, H., Lin, Y.-Q., Kumar, S., McLaughlin, M. M., et al. (2018). A monoclonal antibody targeting amyloid β (Aβ) restores complement factor I bioactivity: Potential implications in age-related macular degeneration and Alzheimer’s disease. *PLoS One* 13, e0195751. doi: [10.1371/journal.pone.0195751](https://doi.org/10.1371/journal.pone.0195751)

Li, S., MacLachlan, T. K., De Luca, A., Claudio, P. P., Condorelli, G., and Giordano, A. (1995). The cdc-2-related kinase, PISSLRE, is essential for cell growth and acts in G2 phase of the cell cycle. *Cancer Res* 55, 3992–3995.

Liu, G.-S., Peshavariya, H., Higuchi, M., Brewer, A. C., Chang, C. W. T., Chan, E. C., et al. (2012). Microphthalmia-associated transcription factor modulates expression of NADPH oxidase type 4: A negative regulator of melanogenesis. *Free Radical Biology and Medicine* 52, 1835–1843. doi: [10.1016/j.freeradbiomed.2012.02.040](https://doi.org/10.1016/j.freeradbiomed.2012.02.040)

Lona-Durazo, F., Mendes, M., Thakur, R., Funderburk, K., Zhang, T., Kovacs, M. A., et al. (2021). A large Canadian cohort provides insights into the genetic architecture of human hair colour. *Commun Biol* 4, 1–12. doi: [10.1038/s42003-021-02764-0](https://doi.org/10.1038/s42003-021-02764-0)

Madjunkova, S., Kocheva, S. A., and Plaseska-Karanfilska, D. (2014). Fanconi anemia founder mutation in Macedonian patients. *Acta Haematol* 132, 15–21. doi: [10.1159/000355191](https://doi.org/10.1159/000355191)

Ogilvie, P., Hofmann, U. B., Bröcker, E. B., and Hamm, H. (2002). [Skin manifestations of Fanconi anemia]. *Hautarzt* 53, 253–257. doi: [10.1007/s001050100236](https://doi.org/10.1007/s001050100236)

Paradisi, A., Tabolli, S., Didona, B., Sobrino, L., Russo, N., and Abeni, D. (2014). Markedly reduced incidence of melanoma and nonmelanoma skin cancer in a nonconcurrent cohort of 10,040 patients with vitiligo. *J Am Acad Dermatol* 71, 1110–1116. doi: [10.1016/j.jaad.2014.07.050](https://doi.org/10.1016/j.jaad.2014.07.050)

Ransohoff, K. J., Wu, W., Cho, H. G., Chahal, H. C., Lin, Y., Dai, H.-J., et al. (2017). Two-stage genome-wide association study identifies a novel susceptibility locus associated with melanoma. *Oncotarget* 8, 17586–17592. doi: [10.18632/oncotarget.15230](https://doi.org/10.18632/oncotarget.15230)

Rao, S., Mondragón, L., Pranjic, B., Hanada, T., Stoll, G., Köcher, T., et al. (2019). AIF-regulated oxidative phosphorylation supports lung cancer development. *Cell Res* 29, 579–591. doi: [10.1038/s41422-019-0181-4](https://doi.org/10.1038/s41422-019-0181-4)

ROS production and mitochondrial dysfunction driven by PU.1-regulated NOX4-p22phox activation in Aβ-induced retinal pigment epithelial cell injury - PMC (n.d.). Available at: <https://www.ncbi.nlm.nih.gov/pmc/articles/PMC7546003/> (Accessed June 10, 2024).

Roth, D. M., Baddam, P., Lin, H., Vidal-García, M., Aponte, J. D., De Souza, S.-T., et al. (2021). The Chromatin Regulator Ankrd11 Controls Palate and Cranial Bone Development. *Front Cell Dev Biol* 9, 645386. doi: [10.3389/fcell.2021.645386](https://doi.org/10.3389/fcell.2021.645386)

Ruggiero, J. L., Dodds, M., Freese, R., Polcari, I. C., Maguiness, S., Hook, K. P., et al. (2021). Cutaneous findings in Fanconi anemia. *J Am Acad Dermatol* 85, 1253–1258. doi: [10.1016/j.jaad.2020.08.047](https://doi.org/10.1016/j.jaad.2020.08.047)

Song, C., Mitter, S. K., Qi, X., Beli, E., Rao, H. V., Ding, J., et al. (2017). Oxidative stress-mediated NFκB phosphorylation upregulates p62/SQSTM1 and promotes retinal pigmented epithelial cell survival through increased autophagy. *PLoS One* 12, e0171940. doi: [10.1371/journal.pone.0171940](https://doi.org/10.1371/journal.pone.0171940)

The regulation of skin pigmentation - PubMed (n.d.). Available at: <https://pubmed.ncbi.nlm.nih.gov/17635904/> (Accessed June 10, 2024).

You, Y., Bai, F., Ye, Z., Zhang, N., Yao, L., Tang, Y., et al. (2018). Downregulated CDK10 expression in gastric cancer: Association with tumor progression and poor prognosis. *Mol Med Rep* 17, 6812–6818. doi: [10.3892/mmr.2018.8662](https://doi.org/10.3892/mmr.2018.8662)

Zhang, W., Yang, M., Zhou, M., Wang, Y., Wu, X., Zhang, X., et al. (2020). Identification of Signatures of Selection by Whole-Genome Resequencing of a Chinese Native Pig. *Front Genet* 11, 566255. doi: [10.3389/fgene.2020.566255](https://doi.org/10.3389/fgene.2020.566255)

Zhong, X., Xu, X., Yu, J., Jiang, G., Yu, Y., Tai, S., et al. (2012). Clinical and biological significance of Cdk10 in hepatocellular carcinoma. *Gene* 498, 68–74. doi: [10.1016/j.gene.2012.01.022](https://doi.org/10.1016/j.gene.2012.01.022)
